# Supplementary material for: Splice-Junction-Based Mapping of Alternative Isoforms in the Human Proteome
Source: Cell Rep. Author manuscript; Available in PMC 2020 Jan 15. (PMC6961840; doi:10.1016/j.celrep.2019.11.026)
Supplement: 2 [file NIHMS1546469-supplement-2.zip › DF1/PXD009737/TestisLysC/TestisLysC_24_MYL6_VVMGPQFFSAEFKEAFQLFDRTGDGK.pdf]

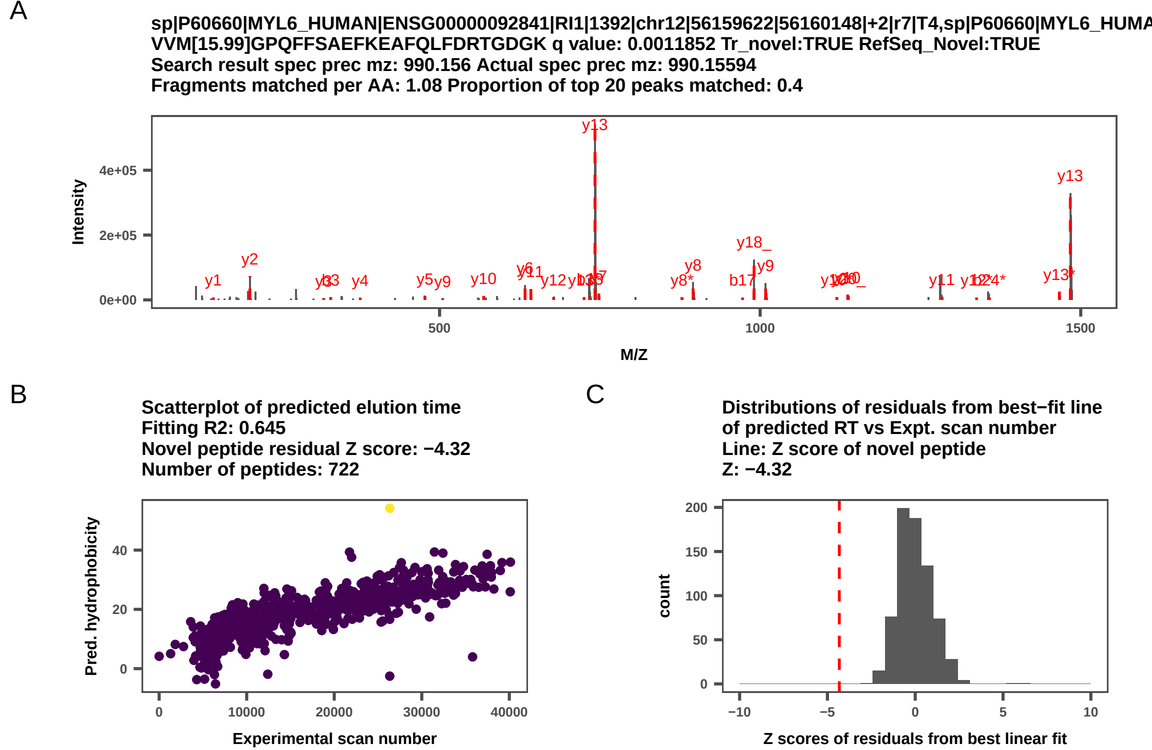
A
sp|P60660|MYL6_HUMAN|ENSG00000092841|RI1|1392|chr12|56159622|56160148|+2|r7|T4,sp|P60660|MYL6_HUMA
VVM[15.99]GPQFFSAEFKEAFQLFDRTGDGK q value: 0.0011852 Tr_novel:TRUE RefSeq_Novel:TRUE
Search result spec prec mz: 990.156 Actual spec prec mz: 990.15594
Fragments matched per AA: 1.08 Proportion of top 20 peaks matched: 0.4
Intensity
4e+05
2e+05
0e+00
y13
y13
y2
y1
y4
y10
y8
y9
y18_
b17
y11
y13*
500
1000
1500
M/Z
B
Scatterplot of predicted elution time
Fitting R2: 0.645
Novel peptide residual Z score: −4.32
Number of peptides: 722
Pred. hydrophobicity
40
20
0
0
10000
20000
30000
40000
Experimental scan number
C
Distributions of residuals from best−fit line
of predicted RT vs Expt. scan number
Line: Z score of novel peptide
Z: −4.32
count
200
150
100
50
0
−10
−5
0
5
10
Z scores of residuals from best linear fit
